# Comparison of Fentanyl and Fentanyl Plus Lidocaine on Attenuation of Hemodynamic Responses to Tracheal Intubation in Controlled Hypertensive Patients Undergoing General Anesthesia

**DOI:** 10.5812/aapm.6442

**Published:** 2013-01-01

**Authors:** Valiallah Hassani, Gholamreza Movassaghi, Vahid Goodarzi, Saeid Safari

**Affiliations:** 1 Department of Anesthesiology, Rasoul-Akram Medical Center, Iran University of Medical Sciences (IUMS), Tehran, Iran; 2 Minimally Invasive Surgery Research Center, Rasoul-Akram Medical Center, Iran University of Medical Sciences (IUMS), Tehran, Iran; 3 Department of Anesthesiology, Hasheminezhad Hospital, Iran University of Medical Sciences (IUMS), Tehran, Iran

**Keywords:** Hemodynamics, Lidocaine, Fentanyl, Intubation, Intratracheal, Drug Stability

## Abstract

**Background:**

Induction of anesthesia and endotracheal intubation often creates a period of hemodynamic instability in hypertensive patients. Endotracheal intubation of the trachea stimulates laryngeal and tracheal sensory receptors, resulting in a marked increase in the elaboration of sympathetic amines.

**Objectives:**

This trial aimed to evaluate and compare the efficacy of fentanyl and fentanyl plus lidocaine in attenuating the hemodynamic responses to laryngoscopy and endotracheal intubation in hypertensive patients.

**Patients and Methods:**

We conducted a prospective, randomized, double-blind trial in 37 patients with hypertension in the Rasoul-Akram Hospital, Tehran, Iran, from March to December 2011. The patients were randomly divided into two groups (fentanyl group and fentanyl plus lidocaine group). The fentanyl group received 2 mcg/kg and the fentanyl plus lidocaine group received 1.5mg lidocaine and 2mcg/kg fentanyl. Hemodynamic variables were recorded at baseline, after giving inductive anesthetic agents, and 1, 3 and 5 minutes after performing endotracheal intubation.

**Results:**

We evaluated 37 patients including 15 males (40.54%) and 22 females (59.46%), with a mean age of 56.08 ± 10.85 years. There were no significant differences between the two groups regarding; heart rate, systolic blood pressure and diastolic blood pressure before induction, 3 minutes before intubation and 1, 3 and 5 minutes after intubation.

**Conclusions:**

Fentanyl and fentanyl plus lidocaine effectively decreased the hemodynamic response to tracheal intubation, however, neither fentanyl nor fentanyl plus lidocaine, could inhibit all hemodynamic responses, moreover fentanyl plus lidocaine was not more effective than fentanyl alone.

## 1. Background

Tracheal intubation may induce; hypertension, tachycardia, and/or arrhythmia. These tracheal responses are mediated by sympathetic responses and are normally well tolerated by normotensive patients. However, induction of anesthesia and endotracheal intubation often produces a period of hemodynamic instability for hypertensive patients and regardless of the level of preoperative blood pressure control, many patients with hypertension display an accentuated hypotensive response to induction of anesthesia, followed by an exaggerated hypertensive response to endotracheal intubation. Endotracheal intubation of the trachea stimulates laryngeal and tracheal sensory receptors, resulting in a marked increase in the elaboration of sympathetic amines (adrenaline and noradrenaline), this sympathetic stimulation results in tachycardia and elevation of blood pressure (1-7). Thus diverse classes of drugs and different techniques such as; local anesthetics, opioids, calcium channel blockers, short acting β-adrenergic blockers, and their combinations have been used to prevent hemodynamic responses induced by laryngoscopy and endotracheal intubation (8-14). The hypothetical background for the use of these methods for laryngoscopy and tracheal intubation is that these adjuvant measures may be able to decrease hemodynamic responses by blocking intense sympathetic discharge caused by stimulation of the upper airway. Fentanyl is a frequently used opioid that joins with hypnotic agents to diminish hemodynamic responses to tracheal intubation (15-17). Furthermore, lidocaine has a suppressive effect on the circulatory responses in patients undergoing laryngoscopy and tracheal intubation (18, 19).

## 2. Objectives

This trial aimed to evaluate and to compare the efficacy of fentanyl and fentanyl plus lidocaine in attenuating hemodynamic responses to laryngoscopy and endotracheal intubation in ASA class ІІ patients (hypertensive patients).

## 3. Patients and Methods

We conducted a prospective, randomized, double-blind trial in 37 patients with hypertension in the Rasoul-Akram Hospital, Tehran, Iran, from March to December 2011. The study protocol was confirmed by the Ethical Committee of the Tehran University of Medical Sciences, furthermore, written informed consent was obtained from all patients. Including criteria comprised; elective surgery with general anesthesia, 65 > age > 20, ASA class ІІ patients (hypertensive patients). Excluding criteria; patients undergoing heart surgery, ASA III or above, CHF (congestive heart failure, arrhythmia, 20 > age > 65 years, problems with intubation, intubation time greater than 15 seconds, contraindications to lidocaine use, and no control of hypertension and/or asthma. The patients' demographic data such as sex and age were recorded, and then patients were randomly divided into two groups (fentanyl group and fentanyl plus lidocaine group). The fentanyl group received 2 mcg/kg and the fentanyl plus lidocaine group received 1.5mg lidocaine and 2mcg/kg fentanyl. Patients received their morning dose of anti-hypertensive medication before surgery. A routine pre-operative check-up was done in all patients and baseline vitals were noted, next patients received normal saline or ringer 5ml/kg in the admission operation room, and then they were oxygenation for three minutes. In the operating room, an intravenous line was started. Patients were attached to the following monitors; ECG, noninvasive blood pressure monitor, pulse oximetery. The baseline values (pre-anesthetic reading) for; mean arterial pressure (MAP) {MAP = DBP + 1/3 (SBP-DBP)}, systolic blood pressure (SBP), diastolic blood pressure (DBP), and heart rate (HR) were recorded. Anesthesia was induced by thiopental given in a 3-5 mg/kg dose with fentanyl, or lidocaine plus fentanyl, next succinylcholine was given in a dose of 1-1.5 mg/kg succinylcholine given approximately 30-60 seconds before endotracheal intubation and induction was confirmed by loss of eyelash reflexes. Fentanyl or lidocaine plus fentanyl was administered three minutes prior to intubation. The hemodynamic variables; MAP), SBP, DBP, and HR were recorded after giving inductive anesthetic agents and trial drugs (before performing endotracheal intubation). Then a laryngoscopy was performed by a professional anesthetist with a standard Macintosh laryngoscope blade and the trachea was intubated with an appropriate size cuffed endotracheal tube and the patient was ventilated with oxygen. Hemodynamic variables; MAP, SBP, DBP, and HR were recorded 3 and 5 minutes after performing endotracheal intubation. We also recorded any possible complications such as; bradycardia (HR < 50), hypotension (SBP < 90), bronchospasm, seizure and rigidity. All results were expressed as mean ± SD. Hemodynamic variables in the present study were analyzed statistically by using a t-test. *P* values ≤ 0.05 were considered significant.

## 4. Results

We evaluated 37 patients including 15 males (40.54%) and 22 females (59.46%) with a mean age of 56.08 ± 10.85 years. There was no significant difference between the two groups regarding HR, SBP and DBP in the patients before induction, three minutes before intubation and 1, 3 and 5 minutes after intubation. Tables 1, 2 and 3 show the hemodynamic variables changes in two groups.

**Table 1.** Mean Standard Deviation and *P* value of Heart Rate in Fentanyl and Fentanyl Plus Lidocaine Groups

Heart Rate Groups	Fentanyl, Mean ± SD	Fentanyl + Lidocaine, Mean ± SD	*P* value
**Before induction**	84.38 ± 13.32	80.25 ± 84	0.33
**3 minutes before intubation**	80.69 ± 11.36	74.83 ± 12.02	0.15
**1 minutes after intubation**	79.23 ± 9.72	73.16 ± 9.97	0.08
**3 minutes after intubation**	73.46 ± 9.19	72.00 ± 11.16	0.68
**5 minutes after intubation**	68.38 ± 11.01	67.50 ± 9.93	0.80

**Table 2.** Mean Standard Deviation and *P* value of Systolic Blood Pressure in Fentanyl and Fentanyl Plus Lidocaine Groups

Systolic Blood Pressure Groups	Fentanyl, Mean ± SD	Fentanyl + Lidocaine, Mean ± SD	*P* value
**Before induction**	149.54 ± 23.54	162.00 ± 25.66	0.15
**3 minutes before intubation**	128.77 ± 16.15	135.04 ± 25.11	0.42
**1 minutes after intubation**	122.38 ± 20.12	123.08 ± 14.84	0.95
**3 minutes after intubation**	116.62 ± 24.54	113.88 ± 24.29	0.74
**5 minutes after intubation**	107.31 ± 21.76	113.42 ± 29.96	0.52

**Table 3.** Mean Standard Deviation and *P* value of Diastolic Blood Pressure in Fentanyl and Fentanyl Plus Lidocaine Groups

Diastolic Blood Pressure Groups	Fentanyl, Mean ± SD	Fentanyl + Lidocaine, Mean ± SD	*P value*
**Before induction**	86.30 ± 10.57	95.41 ± 12.78	0.03
**3 minutes before intubation**	78.53 ± 11.60	79.45 ± 19.05	0.87
**1 minutes after intubation**	73.46 ± 11.34	78.20 ± 13.49	0.28
**3 minutes after intubation**	73.23 ± 14.76	73.83 ± 17.13	0.91

## 5. Discussion

We found that fentanyl and fentanyl plus lidocaine are effective medications on hemodynamic responses (HR, SBP and DBP) decreasing before induction, three minutes before intubation and 1, 3 and 5 minutes after intubation. However, we did not discern any significant difference between the efficacy of the two kinds of medication (fentanyl and fentanyl plus lidocaine). Endotracheal intubation is a stressful noxious force stimuli, it stimulates laryngeal and tracheal sensory receptors, resulting in a marked increase in the expansion of sympathetic amines (adrenaline and noradrenaline), and this increase in the sympathetic amines leads to complications especially in patient with cardiovascular diseases. Responses to endotracheal intubation arise essentially due to sympathetic stimulation causing increases in blood pressure, increases in heart rate and tachyarrhythmia. In normal patients, these responses are significantly high, but they are generally well tolerated, whereas in patients with cardiovascular diseases, many complications may occur like; increases in systolic and diastolic blood pressure, increases in heart rate, tachyarrhythmia, cerebral hemorrhage, left ventricular failure, and in rare conditions, myocardial ischemia (20-25). These hemodynamic responses to intubation in our patients were controlled effectively in the two groups, but adding lidocaine to fentanyl did not increase the hemodynamic stability more than fentanyl alone. In contrast with our study, several previous studies have verified that lidocaine improves intraoperative and postoperative hemodynamic stability by stabilizing the changes in arterial pressure, heart rate and cardiac output. The mechanism behind these beneficial effects of lidocaine on hemodynamic stability is possibly due to; direct myocardial depressant effect, peripheral vasodilating effect and the effect on synaptic transmissions (18, 19). Moreover, another study by Ali et al. in 2010 revealed that pre-treatment with xylocard improves intra- and post-operative hemodynamic stability during laparoscopic surgery without prolonging recovery (26). Our study was in line with some previous studies such as Shin et al. that compared the effects of lidocaine, fentanyl, Nicardipine and Esmolol, on the hemodynamic response during intubation and that study revealed that all of these agents are effective in producing hemodynamic stability (27). Moreover, a study by Levitt et al. found that Esmolol and lidocaine have similar efficacies to attenuate moderate hemodynamic responses to intubation in patients with isolated head trauma (28). Additionally, Malde and Sarode in a 2007 study compared lignocaine and fentanyl efficacy on hemodynamic stability and revealed that lignocaine and fentanyl both attenuated the rise in heart rate; however, fentanyl produced better results. Lignocaine attenuated the rise in blood pressure with intubation while fentanyl inhibits it totally (29). We demonstrated that the two groups of medication were effective on hemodynamic stability but they could not inhibit all hemodynamic responses to intubation. In this case our results were in agreement with Feng et al. (30) and Salihoglu et al. (12) studies that disclosed that lidocaine plus fentanyl were slightly more effective in controlling PR following endotracheal intubation. In conclusion, our study was well designed and we considered factors that could possibly have affected our results, moreover the patients in two groups were matched appropriately. Nevertheless, our results were possibly limited by the fact that we did not monitor the depth of anesthesia. Doses that are entirely based on mcg/kg probably produce different depths of anesthesia in a given population, which may have affected our results. In addition, we firmly declare, that a more profound induction of anesthesia before tracheal tube insertion may also have influenced the results of this study. Fentanyl and fentanyl plus lidocaine are effective in decreasing the hemodynamic response to tracheal intubation, however, neither fentanyl nor fentanyl plus lidocaine could inhibit all hemodynamic responses, furthermore fentanyl plus lidocaine was not more effective than fentanyl alone.
